# Risk Factors for Prolonged Treatment of Whiplash-Associated Disorders

**DOI:** 10.1371/journal.pone.0132191

**Published:** 2015-07-06

**Authors:** Hiroyuki Oka, Ko Matsudaira, Tomoko Fujii, Hiroshi Okazaki, Yukari Shinkai, Yutaka Tsuji, Sakae Tanaka, Ryuichi Kato

**Affiliations:** 1 Department of Medical Research and Management for Musculoskeletal Pain, 22nd Century Medical & Research Center, Faculty of Medicine, University of Tokyo, Tokyo, Japan; 2 Department of Orthopaedic Surgery, Japan Labour, Health and Welfare Organization, Kanto Rosai Hospital, Kanagawa, Japan; 3 Medical Research Center, JA Kyosai Research Institute, Tokyo, Japan; 4 Department of Orthopaedic Surgery, University of Tokyo, Tokyo, Japan; University of Granada, SPAIN

## Abstract

**Objectives:**

Whiplash-associated disorders (WAD) are the most common injuries that are associated with car collisions in Japan and many Western countries. However, there is no clear evidence regarding the potential risk factors for poor recovery from WAD. Therefore, we used an online survey of the Japanese population to examine the association between potential risk factors and the persistence of symptoms in individuals with WAD.

**Materials and Methods:**

An online survey was completed by 127,956 participants, including 4,164 participants who had been involved in a traffic collision. A random sample of the collision participants (n = 1,698) were provided with a secondary questionnaire. From among the 974 (57.4%) respondents to the secondary questionnaire, we selected 183 cases (intractable neck pain that was treated over a period of 6 months) and 333 controls (minor neck pain that was treated within 3 months). Multivariable logistic regression analysis was used to evaluate the potential risk factors for prolonged treatment of WAD.

**Results:**

Female sex, the severity of the collision, poor expectations of recovery, victim mentality, dizziness, numbness or pain in the arms, and lower back pain were associated with a poor recovery from WAD.

**Conclusions:**

In the present study, the baseline symptoms (dizziness, numbness or pain in the arms, and lower back pain) had the strongest associations with prolonged treatment for WAD, although the psychological and behavioral factors were also important. These risk factors should be considered when evaluating patients who may have the potential for poor outcomes.

## Introduction

Whiplash-associated disorders (WAD) are the most common injuries that are associated with car collisions in many Western countries [1] and in Japan [2]. Although the prognosis for WAD is generally favorable, previous studies have found that up to about 20% of patients experience persistent neck pain at 6 months after their injury [3,4]. Unfortunately, this lack of recovery creates personal, economic, and social burdens [1]. To reduce this burden, the number of individuals who develop chronic WAD must be reduced, although it is difficult to predict which patients will experience persistence of their symptoms. However, several prognostic factors have been identified, including sex [5,6], a low level of education [5,6], the severity of the collision [7], expectations of recovery [8], a no-fault claim [7], the presence of dizziness [9], upper extremity numbness or pain [10], and lower back pain [11–13]. Unfortunately, there is no clear evidence regarding the potential risk factors for poor recovery from WAD in the Japanese population. Based on this absence of suitable data, we conducted an online survey of the general Japanese population to identify individuals who had been in a car collision. Using the data from that survey, we examined the associations between the potential risk factors and the persistence of symptoms in individuals with WAD.

## Materials and Methods

### Sources of data

In 2012, we conducted an online survey to assess the prevalence of WAD in the general population. The participants were recruited through an internet research company that has approximately 1.8 million registered Japanese adult volunteers (20–79 years old). The company’s volunteers are representative of the general Japanese population, and were stratified according to sex and age. From among these volunteers, 1,063,083 individuals were randomly selected and invited to participate in this study via an email that contained a unique link to the survey (dated July 1, 2012). Among these invited individuals, only 227,853 were considered effective users, as the research company was unable to exclude the non-users from the invitations due to technical reasons. The participants received points for online shopping as an incentive, and double registration was prevented by reviewing the participant’s e-mail address at the beginning of the survey and disabling the link to the questionnaire at the conclusion of the survey. The initial survey was closed when the number of participants reached 127,956 (July 17, 2012). Thus, the response rate for the invitations was not relevant to this survey. This study’s design was approved by the ethics review board of Kanto Rosai Hospital.

**Fig 1:**
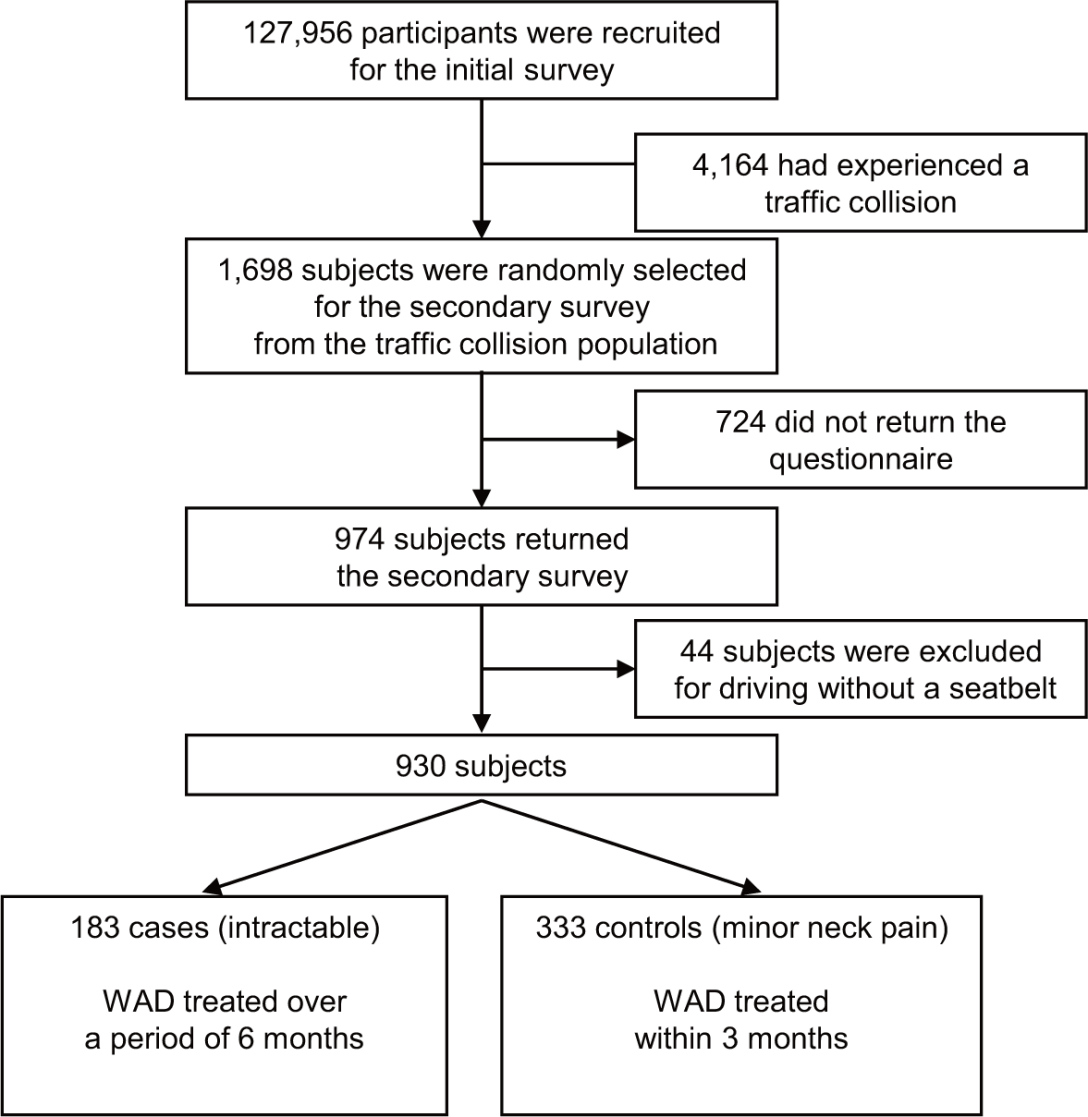
Study flow chart. WAD, whiplash-associated disorders.

All participants completed the original questionnaire, which included items regarding their demographical and social characteristics, as well as any traffic collisions that they had experienced. However, for our analysis we only evaluated the questionnaires from participants who had been in a traffic collision (n = 4,164). From among this sample, 1,698 participants were randomly selected to participate in a secondary survey. Among the 974 respondents (57.4%) for the secondary questionnaire, we excluded 44 participants who were not wearing a seatbelt when the collision occurred, as these participants were likely to have sustained serious injuries. From the 930 remaining subjects, we included 183 participants in the cases group (neck pain that was treated over a period of 6 months) and 333 participants in the control group (minor neck pain that was treated within 3 months) (Fig 1). We defined the self-reported presence of WAD in this study as a response to the internet questionnaire that indicated 1) an obvious instance of an injury that was sustained during a rear-end collision, or 2) an established diagnosis of WAD by a medical doctor.

### Assessment

The questionnaire evaluated socio-demographic data, age, sex, weight, height, smoking, education level (not college, or college), the severity of the collision (high, or other; high severity was defined as the vehicle’s bumpers exhibiting extensive damage after a rear-end collision). Body mass index (BMI; k/m^2^) was calculated using the participant’s self-reported weight and height. Expectations of recovery were evaluated by asking “Do you expect that your neck pain will be a problem in the next 3 months?”, using response categories of “No”, “Possibly”, “Probably”, and “Definitely”. Poor expectations of recovery were defined as answers of “Probably” or “Definitely”. We also used the question “Did you have any fault in this accident?” to identify participants with a “victim mentality” (i.e., an answer of “no”). The presence of dizziness (yes/no) was evaluated using the question “Did you have any dizziness in the week after this accident?”, and numbness or pain in the arms was evaluated using the question “Did you have any numbness or pain in your arms in the week after this accident?” Lower back pain was defined as pain that lasted for >1 day in the area between the lower costal margin and the gluteal folds, regardless of any accompanying radiating pain, and that was not associated with febrile illness, menstruation, or pregnancy [14].

### Statistical analysis

The preliminary survey was administered to 10,000 participants for sample size estimation. Our preliminary study revealed that 16 of the 10,000 participants were assigned to the case group. 2) As our dependent variable was binary, we decided to use logistic regression analysis, because we needed a 1:2 case:control ratio. One guideline has suggested that the accurate estimation of discriminant function parameters requires a sample size with at least 20 cases for each independent variable in the logistic regression [15]. Therefore, based on this guideline and our 10 predictor variables, we required 200 cases for our analysis. Thus, the survey was closed at approximately 125,000 participants, although slightly more than 125,000 participants were included, due to technical reasons.

We compared the characteristics of the cases and controls using the chi-square test for categorical variables, and the one-factor analysis of variance for numerical variables. Age, sex, BMI, smoking, education level, severity of collision, poor expectation of recovery, victim mentality, dizziness, numbness or pain in arms, and lower back pain have previously been identified as risk factors for a poor recovery from WAD [5–13]. Therefore, we entered these variables into the multivariable logistic regression model, in order to adjust for potential confounding. The Variance Inflation Factor (VIF) was used to check for multicollinearity in the model. All statistical tests were performed at a significance level of 0.05 (two-sided), and were not adjusted for multiple testing. All data analyses were performed using SAS software (version 9.1.3, SAS Institute Inc., Cary, NC).

## Results

Table 1 shows the demographic characteristics of the participants. When we compared the case and control groups, we observed significant differences in the severity of the collision, poor expectations of recovery, dizziness, upper extremity numbness or pain, and lower back pain. However, no significant differences were observed for age, sex, BMI, smoking, and a low level of education.

**Table 1:** 

	Cases (n = 183)	Controls (n = 333)	p-value
Age, years	44.8 ± 10.3	45.3 ± 11.7	0.6218
Sex, male/female	124/59	242/91	0.2397
BMI, kg/m^2^	23.4 ± 4.0	23.0±3.7	0.1971
Smoking (%)	74 (36.6)	128 (38.4)	0.6563
Education level: not college (%)	57 (31.2)	99 (29.7)	0.7373
Severity of collision: high (%)	131 (71.6)	159 (47.9)	<0.0001
Poor expectation of recovery (%)	90 (49.2)	41 (12.3)	<0.0001
Victim mentality (%)	150 (83.0)	253 (76.0)	0.1154
Dizziness (%)	120 (65.6)	94 (28.2)	<0.0001
Numbness or pain in arm (%)	149 (81.4)	170 (51.1)	<0.0001
Low back pain (%)	113 (61.2)	74 (22.2)	<0.0001

BMI, body mass index.

Table 2 shows the results from the univariate logistic regression analysis for a poor recovery from WAD. Based on the results of this analysis, we found that female sex, the severity of the collision, poor expectations of recovery, victim mentality, dizziness, numbness or pain in the arms, and lower back pain were significantly associated with a poor recovery from WAD. Table 3 shows the results from the multivariable logistic regression analysis, after adjusting for the various confounding factors. The VIF values for age, sex, BMI, smoking, education level, severity of collision, poor expectation of recovery, victim mentality, dizziness, numbness or pain in arms, and lower back pain were 1.12, 1.12, 1.14, 1.03, 1.19, 1.17, 1.16, 1.26, 1.23, and 1.24, respectively. However, none of the VIF values exceeded 10, which indicates that there was no collinearity in the model [16]. Based on the results of this model, we found that female sex, the severity of the collision, poor expectations of recovery, victim mentality, dizziness, numbness or pain in the arms, and lower back pain were significantly associated with a poor recovery from WAD.

**Table 2:** 

	Odds ratio	95% CI	p-value
Age, +1 year	1	0.99–1.02	0.6209
Female (vs. male)	1.26	0.85–1.87	0.2417
BMI (+1 kg/m^2^)	0.97	0.92–1.02	0.1983
Smoking	0.92	0.64–1.33	0.6566
Education level: not college	1.06	0.72–1.58	0.7376
Severity of collision: high	2.76	1.88–4.08	<0.0001
Poor expectation of recovery	6.89	4.48–10.76	<0.0001
Victim mentality	1.44	0.92–2.28	0.1114
Dizziness	4.84	3.30–7.17	<0.0001
Numbness or pain in arms	4.2	2.76–6.54	<0.0001
Lower back pain	5.65	3.82–4.82	<0.0001

CI, confidence interval; BMI, body mass index.

**Table 3:** 

	Odds ratio	95% CI	p-value
Age, +1 year	1	0.98–1.03	0.7577
Female (vs. male)	1.83	1.07–3.17	0.0283
BMI (+1 kg/m^2^)	1.07	0.99–1.14	0.0576
Smoking	0.95	0.58–1.57	0.8515
Education level: not college	1.11	0.67–1.85	0.6819
Severity of collision: high	1.97	1.19–3.30	0.0086
Poor expectation of recovery	4.47	2.68–7.53	<0.0001
Victim mentality	3.37	1.76–6.67	0.0002
Dizziness	3.12	1.93–6.00	<0.0001
Numbness or pain in arms	2.56	1.51–4.40	0.0004
Lower back pain	4.77	2.91–7.94	<0.0001

CI, confidence interval; BMI, body mass index.

## Discussion

To the best of our knowledge, this is the first study to examine the risk factors that are associated with a prolonged recovery among Japanese patients with WAD. Our final model identified seven risk factors (female sex, the severity of the collision, poor expectations of recovery, victim mentality, presence of dizziness, numbness or pain in the arms, and lower back pain); all of these factors have previously been reported to be independent prognostic factors for recovery from WAD [5–13].

Interestingly, it is not clear which sex is an independent risk factor for poor recovery from WAD, as several studies have reported that female sex was an independent predictor, while others have reported that male sex was an independent predictor. In addition, previous studies have reported that a low level of education was significantly related to a poor recovery [5,6]. However, in the present study, education level was not a significant risk factor for a poor recovery from WAD. Unfortunately, the reasons for these discrepancies between our findings and those of the previous studies are not clear, although they may be related to differences in the populations that were studied.

We also observed that the severity of the collision was an important risk factor for poor recovery from WAD. In this context, a whiplash injury occurs when the force of a rear-end collision “whips” the cervical spine beyond its normal range of motion. Therefore, it is logical that severe car crashes can cause serious damage to the musculoskeletal system, which can result in a poor recovery.

After adjusting for the relevant confounders, such as socio-demographic characteristics and symptoms, we observed that poor expectations of recovery and victim mentality were significant risk factors for a poor recovery. Similarly, previous studies have reported that expectations for recovery were an important factor in the prognosis for WAD recovery [5]. Therefore, in addition to understanding these injuries and their clinical symptoms, it is also important to understand the patient’s perception of recovery, in order to adequately treat WAD. Furthermore, victim mentality is an aspect of the patient’s perception, and may affect their expectations for recovery. This finding indicates that psychological factors have prognostic value for evaluating the risk of prolonged recovery from WAD.

A previous study has reported that dizziness, numbness in the arms, and lower back pain did not decrease within 6 months after the accident, although many other symptoms were transient [13]. Similarly, we observed that these symptoms (dizziness, numbness, and lower back pain) were independent risk factors for a prolonged recovery from WAD. Therefore, it appears that these symptoms are more common in severe cases, which are less likely to experience recovery within 6 months. Furthermore, dizziness, numbness, and lower back pain are known as somatic symptom, and patients who have chronic whiplash also report elevated levels of somatic symptoms in body areas that were not affected by their neck trauma [17, 18]. In this context, the symptoms of functional somatic syndromes are very similar to those of somatization disorder, and the two conditions are thought to be closely related [19–21]. Thus, it is important to consider these signs and symptoms when following-up patients who have experienced whiplash. Furthermore, although the baseline symptoms (dizziness, numbness, and lower back pain) had the strongest associations with prolonged treatment for WAD, the psychological and behavioral factors were also important, and these risk factors should also be considered when evaluating patients who have experienced whiplash.

This study has several limitations. First, due to the cross-sectional design, inferences cannot be made regarding the causality of the relationships. Second, the sample was selected from among internet research volunteers, who may not be representative of the general population of internet users. Third, compared to the general population, our sample contained a higher proportion of people who were living in large cities and who had completed university-level or graduate-level education [22]. Fourth, we surveyed the respondents after their traffic collisions, and it is plausible that some reported symptoms may have been preexisting, rather than caused by the traffic collision. Furthermore, there are other important factors that can affect recovery from WAD, such as coping styles, previous traffic injuries, comorbidities, somatic and psychological pre-injury health, pain intensity and disability, injustice perception, depression and pain-related emotions, social support, personality traits, and post-traumatic stress symptoms. However, these factors were not included because we needed to evaluate the information from at the time of injury as a prognostic factor. Therefore, recall bias may be present, given the interval between the injury and the administration of the validated questionnaires. In addition, we attempted to ensure that the full questionnaire could be completed in 10 min, in order to obtain complete data from the respondents. Unfortunately, the effect of this selection bias on our findings would be difficult to address. Despite these limitations, this study provides useful insight for medical and public health practitioners who treat patients who have experienced whiplash.
